# Association of microbial dynamics with urinary estrogens and estrogen metabolites in patients with endometriosis

**DOI:** 10.1371/journal.pone.0261362

**Published:** 2021-12-16

**Authors:** Nhung Le, Melissa Cregger, Veronica Brown, Julio Loret de Mola, Pamela Bremer, Lyn Nguyen, Kathleen Groesch, Teresa Wilson, Paula Diaz-Sylvester, Andrea Braundmeier-Fleming

**Affiliations:** 1 Department of Medical Microbiology, Immunology and Cell Biology, Southern Illinois University School of Medicine, Springfield, Illinois, United States of America; 2 Biosciences Division, Oak Ridge National Laboratory, Oak Ridge, Tennessee, United States of America; 3 Department of Ecology and Evolutionary Biology, University of Tennessee, Knoxville, Tennessee, United States of America; 4 Division of Biology, University of Tennessee, Knoxville, Tennessee, United States of America; 5 Department of Obstetrics and Gynecology, Southern Illinois University School of Medicine, Springfield, Illinois, United States of America; 6 Center for Clinical Research, Southern Illinois University School of Medicine, Springfield, Illinois, United States of America; Washington State University - Spokane, UNITED STATES

## Abstract

Endometriosis is an estrogen dependent gynecological disease associated with altered microbial phenotypes. The association among endogenous estrogen, estrogen metabolites, and microbial dynamics on disease pathogenesis has not been fully investigated. Here, we identified estrogen metabolites as well as microbial phenotypes in non-diseased patients (*n* = 9) and those with pathologically confirmed endometriosis (P-EOSIS, *n* = 20), on day of surgery (DOS) and ~1–3 weeks post-surgical intervention (PSI). Then, we examined the effects of surgical intervention with or without hormonal therapy (OCPs) on estrogen and microbial profiles of both study groups. For estrogen metabolism analysis, liquid chromatography/tandem mass spectrometry was used to quantify urinary estrogens. The microbiome data assessment was performed with Next generation sequencing to V4 region of 16S rRNA. Surgical intervention and hormonal therapy altered gastrointestinal (GI), urogenital (UG) microbiomes, urinary estrogen and estrogen metabolite levels in P-EOSIS. At DOS, 17β-estradiol was enhanced in P-EOSIS treated with OCPs. At PSI, 16-keto-17β-estradiol was increased in P-EOSIS not receiving OCPs while 2-hydroxyestradiol and 2-hydroxyestrone were decreased in P-EOSIS receiving OCPs. GI bacterial α-diversity was greater for controls and P-EOSIS that did not receive OCPs. P-EOSIS not utilizing OCPs exhibited a decrease in UG bacterial α-diversity and differences in dominant taxa, while P-EOSIS utilizing OCPs had an increase in UG bacterial α-diversity. P-EOSIS had a strong positive correlation between the GI/UG bacteria species and the concentrations of urinary estrogen and its metabolites. These results indicate an association between microbial dysbiosis and altered urinary estrogens in P-EOSIS, which may impact disease progression.

## Introduction

Endometriosis is an estrogen-dependent disease characterized by the establishment of ectopic endometrial implants in the peritoneal cavity and elsewhere in the body [1–5]. In the United States, this disease affects over 12% of reproductive-aged women, and 45% of women with endometriosis report sub-fertility or infertility [6, 7]. Current hormonal treatment regimens reduce endometriosis-associated pain and lesion progression by lowering local and systemic estrogen levels and/or suppressing estrogen receptors thus impacting estrogen signaling; however, hormonal treatment does not eradicate the presence of endometriotic tissue [8]. Additionally, after cessation of hormonal therapy, recurrence of symptoms is common (30–35% of women with mild endometriosis and 60–70% of women with severe endometriosis) [9, 10]. Laparoscopic surgical excision/ablation of endometrial lesions temporarily relieves pain, reduces the abdominal inflammatory environment and can restore fertility [11, 12]. Yet, 25% of patients with surgical lesion removal have pain recurrence and often require additional surgical intervention within 2 years [11, 13].

Attachment and growth of endometriotic lesions is promoted through estrogen signaling. Ectopic endometriotic lesions and eutopic endometrial tissue in P-EOSIS express high levels of P450 aromatase, which induces the aromatization of estrone (E1) from adrenal or ovarian androstenedione. E1 is then converted to the most potent estrogen, 17β-estradiol (E2), by 17β-hydroxysteroid dehydrogenase. E1 and E2 bind and activate receptors ERα and ERβ, which are highly expressed in both ectopic and eutopic endometrial tissues. These trophic factors of estrogen then facilitate attachment, development and maintenance of endometriotic lesions in a self-perpetuating manner as these factors increase local production of estrogens due to molecular aberrations in steroidogenesis. Additionally, estrogen signaling also induces an inflammatory peritoneal environment via activation of peritoneal macrophages and production of inflammatory cytokines [14–19]. To date, there is little understanding regarding the role of estrogen metabolites in P-EOSIS, and how specific microbes may influence the production of estrogen and the function of estrogen metabolites in P-EOSIS.

Systemic inflammation is known to alter microbial community dynamics in the mucosal tissues of P-EOSIS [20]. The presence of specific bacterial species (*Lactobacillus*, *Bifidobacterium infantis*, *etc*.) in the urogenital (UG) tract protects mucosal epithelial cells by creating an unsuitable environment for pathogens to survive via the production of lactic acid which lowers the pH of the vaginal environment [21]. Hormones in women play a critical role in changing the UG microbiome. The loss of estrogen during menopause can decrease the relative amounts of commensal species, with a subsequent rise of multiples gynecological diseases [22]. In addition, gut bacteria are known to regulate steroid metabolism through the enterohepatic recirculation pathway, which is critical for cholesterol metabolism and downstream regulation of metabolic processes [23, 24]. In the bile, GI microflora then influence human estrogen metabolism by hydrolyzing most of the conjugated estrogens [25]. In P-EOSIS, the elevation of circulating bioactive estrogens could trigger development and maintenance of endometriotic lesions. Therefore, alterations in microbial communities in P-EOSIS may impact estrogen production. Thus, identifying microbial communities that are present in a healthy individual and understanding how these dynamics have shifted in P-EOSIS may provide a powerful diagnostic tool for this disease [20].

We hypothesized that P-EOSIS have altered gastrointestinal/urogenital microbial communities and aberrant estrogen levels that are distinct from those of non-diseased patients. Further, surgery and/or hormonal therapy will temporarily restore the microbiome and estrogen levels of P-EOSIS. To test these hypotheses, we identified estrogen metabolites as well as microbial phenotypes in non-diseased patients and those with pathologically confirmed endometriosis. Then, we examined the effects of surgical intervention with or without hormonal therapy on estrogen and microbial profiles of both study groups.

## Materials and methods

### Study subjects

Subjects were enrolled and samples were collected according to standard operating procedures approved by the local Institutional Review Board (Springfield Committee for Research Involving Human Subjects) under the protocol #14–220. All subjects were recruited from the Department of Obstetrics & Gynecology of Southern Illinois University (SIU) School of Medicine. Women (aged 18–51) undergoing laparoscopy/laparotomy for the investigation of unexplained pelvic pain, suspicion of or known to have endometriosis who provided informed consent were enrolled in the experimental group (P-EOSIS). Surgical confirmation and staging of endometriosis were completed by a board certified Reproductive Endocrinology and Infertility Specialist. A second group of women scheduled for laparoscopy/laparotomy/hysterectomy for either benign uterine or ovarian indications (i.e. Mullerian anomalies, blocked fallopian tubes/hydrosalpinx, fibroids, endometrial polyps, abdominal/pelvic adhesions, etc.) or multi-parity (i.e. tubal ligation) were enrolled in the control group (CON). Subjects in either cohort were eligible if currently utilizing monophasic hormonal contraception (oral contraceptive pills: OCPs) or not utilizing OCPs as long as their exogenous hormonal suppression (or lack thereof) was consistent across study time points. Exclusion criteria: pregnancy, visual evidence of *or* current treatment for cervical or pelvic infection/inflammatory disease, menopausal or peri-menopausal status, and the current usage of an intra-uterine device. Samples from a total of 29 (P-EOSIS = 20; CON = 9) subjects were collected and analyzed. Each subject’s electronic medical record was thoroughly reviewed for general health, age, race, parity, socioeconomic status, antibiotic use, nutrition/diet information and use of exogenous hormones as variables to be included in our analysis (Table 1). Consented subjects also completed a standardized microbiome questionnaire including specific topics such as personal hygiene, social and sexual history to provide insights into factors influencing the subject’s GI and UG microbiome.

**Table 1:** Demographic of study population.

		EOSIS	Control	P-value
		(n = 20)	(n = 9)	
Age (Mean ± S.E.M.)		32.5 ± 1.1	32.6 ± 2.0	0.48
Race	Caucasian	18	9	0.24
	Hispanic	1	0	
	Other/unspecified	2	0	
BMI (Mean ± S.E.M.)		26.5 ± 1.5	28.1 ± 2.4	0.3
Hormonal therapy	Yes	10	5	0.9
	No	10	4	
Disease Stage (r-ASRM* score)	Stage 1 (1–5)	5	N/A	N/A
	Stage 2 (6–15)	3		
	Stage 3 (16–40)	4		
	Stage 4 (>40)	8		

BMI: body mass index.

ASRM: American Society Reproductive Medicine.

P-values calculated using t test and chi-square test.

### Sample collection and preparation

Sample collection methods were performed by trained medical professionals to obtain standardized specimens from the anal canal and vagina per the Human Microbiome Project protocol [26]. Urine, fecal and vaginal swab samples were collected at two different time points: the *day of surgery (DOS)* and at the *1–3 weeks postoperative appointment (PSI)*.

Urine samples (10–50 ml) without preservative were centrifuged to remove cellular debris. Fecal and vaginal swabs were immediately placed separately into 1 ml sterile Ca^2+^/Mg^2+^ free phosphate-buffered saline and stored at −80°C until DNA extraction was performed.

### Urinary estrogen metabolites

Liquid chromatography-tandem mass spectrometry (LC-MS/MS) was used to quantify estrogen metabolites. Levels of estrogen metabolites were normalized to urinary creatinine. Spectrometry was performed at the University of Illinois Metabolomics Core. Urine samples were briefly derivatized with dansyl chloride and analyzed by LC-MS/MS [27]. Urine specimens (1 mL) were spiked with 10 μL of 1 ng/ml of 5 deuterated estrogens and extracted by vortexing with 5 mL dichloromethane. Extracts were transferred to fresh tubes and dried using a vacuum centrifuge. Residues were dissolved with 50 μL of 1 mg/mL dansyl chloride in acetonitrile by brief vortexing, followed by the addition of a 50 μL 0.1M sodium bicarbonate buffer (9.0 pH) and heated for 5 minutes at 60ºC. The reaction product was transferred to Eppendorf vials and centrifuged for 5 minutes at 20,000 x g prior to the transferring of clear supernatant to LC inserts for analysis. The resulting dansyl-estrogen derivatives were analyzed using a Waters Aquity UPLC Separations Module coupled to a Waters Quattro Premier XE Mass Spectrometer and exported to Excel for additional normalization as required. Area under the curve (AUC) ratios of standard versus deuterated estrogens were used as a response, and a curve of 6 calibration standards (5–1000 pg/ml in CSS, extracted as noted above) was used for quantification. The total metabolite concentration for each of the three estrogen metabolic pathways was determined from the integrated AUC for each respective metabolite that had been normalized to the deuterated standard [28]. The 15 estrogen and estrogen metabolites that were detected included: estrone (E1) with its 2-hydroxylestrone-3methyl ether, 2-, 4-, 16α-hydroxyl, and 2-, 4-methoxyl metabolites; 17β-estradiol (E2) with its 2-hydroxyl, 2-, 4-methoxyl metabolites; estriol (E3), 16-, 17-epiestriol and 16-ketoestradiol.

### Microbial community analysis

DNA extraction was performed on fecal specimens and vaginal samples using a MoBio PowerSoil DNA Isolation kit (Qiagen, Carlsbad, CA). After extraction, the DNA stock concentration was measured using a Qubit^™^ dsDNA BR (Broad-Range) Assay Kit (Q32850; Invitrogen).

#### 16S rRNA gene amplification and sequencing

Bacterial sequencing targeted the V4 region of the 16S rRNA gene (archaeal/bacterial) with a two-step polymerase chain reaction (PCR) approach using the Illumina Nextera XT sequencing protocol. The forward and reverse primer mixture was modified and amplified as previously described, with four variants of 515F and one 806R primer modified for the Illumina MiSeq platform [29]. The thermal cycler conditions for the primary PCR were: 3 minutes at 95ºC followed by 35 cycles of 95ºC for 30 seconds, 55ºC for 30 seconds and 72ºC for 30 seconds with a 5 minute final extension at 72ºC. The PCR products were purified with Agencourt Ampure XP beads (Beckman Coulter, Indianapolis, IN) and each sample was then individually labeled with a unique set of forward and reverse indexes through a second PCR. The secondary index PCR cycle was the same as above, but with only 8 cycles, and the resulting product was again purified with Agencourt Ampure XP beads. These DNA amplicons were normalized, pooled to a final loading concentration of 4 pM with 20% PhiX spike-in and sequenced bi-directionally 250 bases using v2 reagents on the MiSeq platform (Illumina, San Diego, CA) at the University of Tennessee Genomics Core.

#### Sequence bioinformatics analysis

Data were quality filtered and processed using QIIME2 [30]. First, paired end reads were merged with a Phred quality threshold of Q30; then a quality assessment was performed by specific filtering conditions in accordance with QIIME2 quality control process (Trim and truncate primers: trim-left-forward and reverse = 10, trunc-len-forward and reverse = 249). Exact sequence variants (ESVs) were clustered using the DADA2 algorithm [30] and aligned to the Greengenes-reference v. 13.8 database for archaea/bacteria. Finally, artifact sequences or host contamination (i.e. mitochondria, chloroplast or eukaryote) were filtered out.

#### Sequencing statistics

A total of 430,259 sequences were obtained after quality filtering and sequence processing. Across samples, the minimum and maximum number of sequences obtained were 13,501 and 408,567, respectively. The average number of sequences per sample was 81,289 for fecal samples and 95,638 for vaginal samples. The dataset was rarified to 13,501 sequences per sample to account for variation in sequencing depth, no samples were removed. At 13,501 sequences per sample, rarefaction curves plateaued indicating sufficient sequencing for the discovery and investigation of the gut/vagina microbial communities (S1 Fig).

### Statistical analysis

Power analysis (using G Power) shows that we can detect a 2-fold difference between our groups with 80% probability for a sample set of controls (n = 9) and endometriosis (n = 20). Our low patient number was supported by the use of repeated measures statistical tests on samples from DOS to PSI with one way ANOVA. Alpha diversity and evenness were estimated for each sample using Simpson’s evenness measure E, Simpson’s index diversity, and Faith’s PD (phylogenetic diversity metrics) calculated in QIIME2. Microbiome alpha-diversity comparisons among CON and P-EOSIS, and medical interventions (surgery and OCPs) were assessed by ANOVA with estrogen and its metabolites as variables (Qimme2R and phyloseq packages). A Principal coordinate analysis (PCoA) was performed to obtain principal coordinates and visualize multidimensional data using phyloseq package, tidyverse package, and ggplot2 package in R (version 2.15.3). Beta diversity (diversity between samples) on both weighted and unweighted UniFrac was conducted to compare dissimilarity between samples via QIIME2. A constrained analysis of principal coordinates ([CAP], capscale function in vegan package) was calculated for bacteria in GI/UG samples with estrogen metabolites included as predictor variables. Variation in community composition among samples was visualized via a non-metric multidimensional scaling plot (NMDS) based on weighted and unweighted Unifrac with phyloseq package. Statistical differences in community composition were assessed using PERMANOVA in QIIME2 with 999 permutations to measure factors driving bacterial community composition [31, 32]. For taxon abundance, raw counts were retained and normalized by clr transformation; one-way ANOVA was used to study how endometriosis, surgical intervention and hormonal therapy influenced taxon abundances. Pearson correlations were performed using QIIME to assess the relationships between the GI/UG diversity and estrogen variables in all samples.

A non-parametric Mann Whitney U test in GraphPad Prism was performed for each estrogen metabolite, trait measurement (control, P-EOSIS), and medical interventions (surgery and OCPs) to determine the effects of the disease, surgery intervention and/or hormonal therapy on the level of estrogen and its metabolites. All values were considered statistically significant if p < 0.05.

## Results

A total of 29 subjects were included in this study. To establish clinical severity of endometriosis, we utilized the 1996 Revised Classification of Endometriosis from the American Society of Reproductive Medicine [33]. We classified our subjects into four stages as follows: stage 1 (minimal) with a score of 1 to 5, stage 2 (mild) with a score of 6 to 15, stage 3 (moderate) with a score of 16 to 40 and stage 4 (severe) with a score > 40 [34, 35]. Endometriosis was minimal in 5 subjects, mild in 3 subjects, moderate in 4 subjects and severe in 8 subjects (Table 1). All subjects were grouped based on hormonal therapy use at DOS and PSI time points. The type of monophasic hormonal therapy used was consistent for each subject at both time points. Monophasic combined oral contraceptive pills (OCPs) with 1 mg of norethindrone and 35 micrograms of ethinyl estradiol were used by 5 CON subjects and 11 P-EOSIS. We found no significant differences in age, race, BMI nor OCPs utilization between CON and P-EOSIS (Table 1).

### Estrogen metabolites in urine of patients with endometriosis

**Fig 1:**
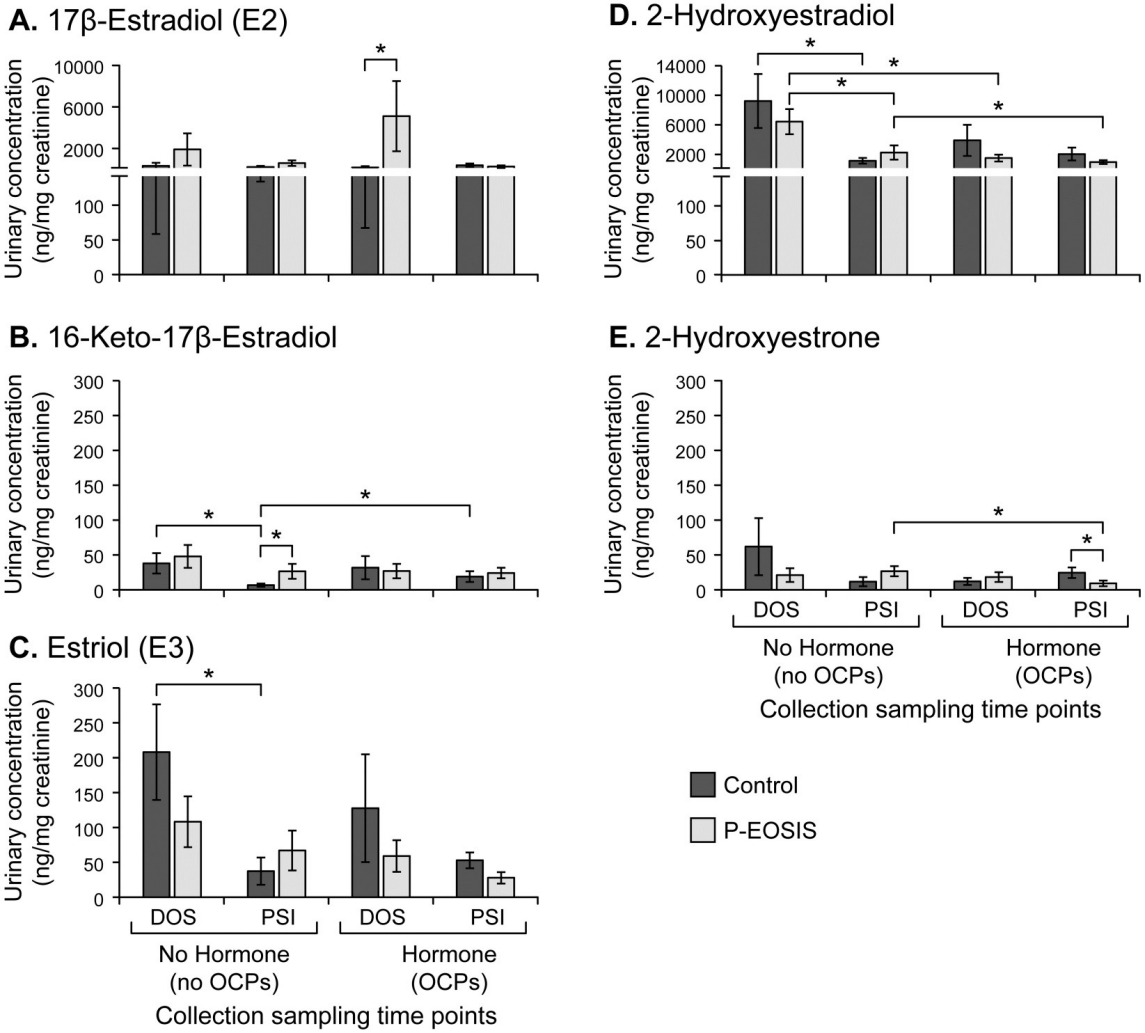
Urinary estrogen metabolites levels in control subjects (control) or patients with endometriosis (P-EOSIS). All subjects were either without hormonal therapy (left panel; control = 4; P-EOSIS = 10) or on hormonal therapy (right panel; control = 5; P-EOSIS = 10) at the time of surgery (DOS) and post-surgery intervention (PSI). Levels of estrogen metabolites were normalized to urinary creatinine. **A**. 17β-estradiol. **B**. 16-Keto-17β-Estradiol. **C**. Estriol. **D**. 2-Hydroxyestradiol. **E**. 2-Hydroxyestrone. * indicates significant difference between groups, Mann-Whitney test, *P-value <0*.*05*, error bars indicate SEM.

To determine the effects of endometriosis, hormonal and surgical treatments on estrogen metabolism in P-EOSIS, urine samples were analyzed by using a highly specific LC-MS/MS for urinary estrogens and estrogen metabolites: estrone (**E1**); 17β-Estradiol (**E2**); 3 catechol estrogens; 5 estrogen metabolites in the 16α pathway, including estriol (**E3**); and 5 methoxy estrogens (Fig 1). The concentrations of three of these metabolites were significantly affected by the presence of the disease (Fig 1A, 1B & 1E, significant differences denoted by asterisks). P-EOSIS had increased E2 when taking OCPs (Fig 1A) and increased 16-keto-17β-estradiol when not utilizing OCPs at PSI (Fig 1B; p = 0.007); whereas levels of 2-hydroxylestrone decreased in P-EOSIS treated with OCPs at PSI (Fig 1E; p = 0.008).

Next, surgical intervention altered 4 estrogen metabolites in both study groups (CON, P-EOSIS) (Fig 1B–1D). The concentrations of 16-keto-17β-estradiol, E3, and 2-hydroxyestradiol decreased after surgery in CON subjects not receiving OCPs (Fig 1B–1D). Similarly, surgical intervention alone (no OCPs) resulted in decreased levels of 2-hydroxyestradiol in P-EOSIS (Fig 1D; p = 0.02). Interestingly, after surgical intervention E2 levels in P-EOSIS decreased to reach levels similar to those observed in CON subjects (Fig 1A).

Finally, hormonal therapy also affected the concentrations of estrogen and its metabolites in CON and P-EOSIS (Fig 1B, 1D & 1E). OCP use resulted in increased levels of 16-keto-17β-estradiol in CON (Fig 1B; p = 0.04), but decreased levels of 2-hydroxyestradiol (metabolite of E2, Fig 1D; p = 0.03) and 2-hydroxylestrone (metabolite of E1, Fig 1E; p = 0.04) in P-EOSIS. These results indicated that excreted urinary parent estrogens (specifically 17β-estradiol and low/undetected estrogen metabolites from 2-OH, 4-OH and 16-OH pathways) were clearly altered in P-EOSIS. Most notably, our data indicates that in all patients studied, conversion of 2-OH estrone to 2-OH estradiol was favored as evident by the higher urinary levels of 2-OH-estradiol compared with 2-OH-estrone. Additionally, estradiol and metabolites of the 2-OH pathway exhibited the greatest change contingent upon hormonal therapy and surgical interventions in both study groups.

### Differences in bacterial community composition between patients with endometriosis and controls in the GI/UG through time

**Fig 2:**
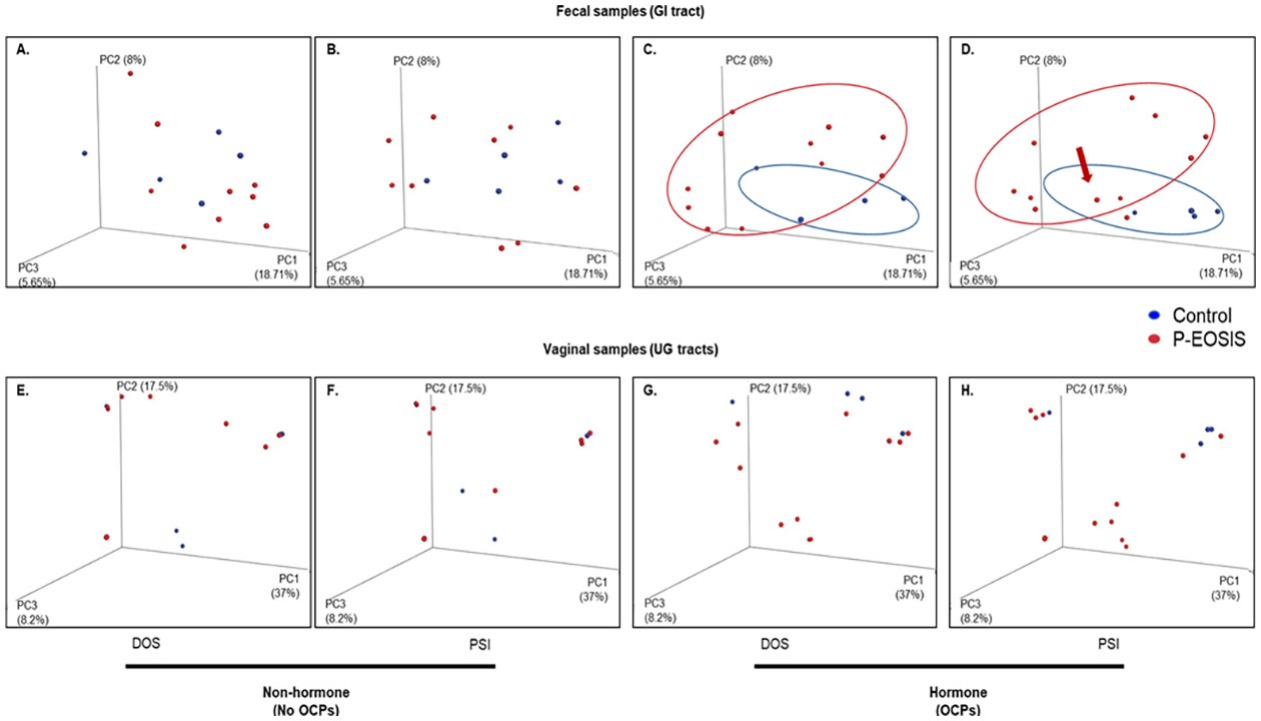
Principal Coordinates Analysis (PCoA). PCoA shows the distribution of GI (fecal samples) and UG (vaginal samples) bacterial communities on the day of surgery (DOS) and post-surgery intervention (PSI) for patients with endometriosis (P-EOSIS) and controls subjects (control) that were receiving or not receiving hormonal treatment (OCPs). PC1, PC2 and PC3 were plotted on the x, y and z axes. **A., E**. (DOS), **B., F**. (PSI): Non-hormone (fecal: n = 9, vagina: n = 14). **C., G**. (DOS), **D., H**. (PSI): Hormone (fecal: n = 20, vagina: n = 15).

**Fig 3:**
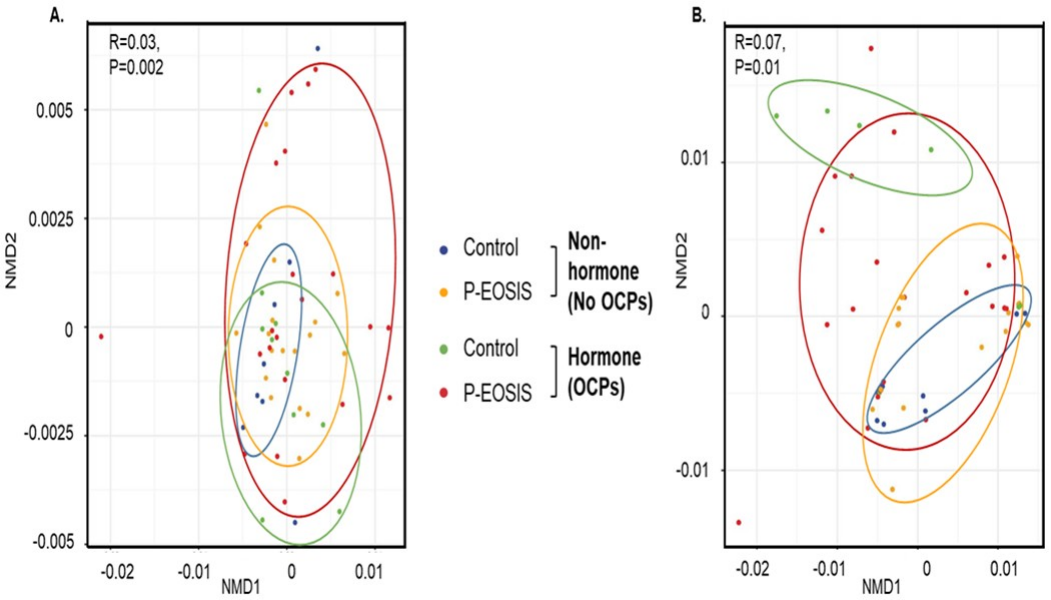
Non-metric dimensional scaling ordination of beta diversity analysis using UniFrac for GI/UG microbial composition. Patients with endometriosis (P-EOSIS) and control subjects (control) were either receiving or not receiving hormonal treatment (OCPs). **A**. Gastrointestinal tract. **B**. Urogenital tract. (No-OCPs samples: fecal: n = 9; vaginal swabs: n = 8; OCPs samples: fecal: n = 20; vaginal swabs: n = 20).

To compare differences in microbial composition among all groups by sample types (fecal and vaginal) we performed beta diversity analyses with weighted and unweighted UniFrac distance metrics that use phylogenetic information. At DOS, we found that GI bacterial communities were similar between P-EOSIS and CON subjects not receiving OCPs (Fig 2A) but significantly differed when OCPs were used (Fig 2C; unweighted p = 0.001, weighted p = 0.029). After surgery, the GI bacterial communities of P-EOSIS who used OCPs became more similar to those of CON subjects (Fig 2D **with arrow**, unweighted p = 0.165, weighted p = 0.424). This may suggest that OCP use alters gut microbial communities and subsequent surgical intervention could restore the gut microbial communities of P-EOSIS. For vaginal microbial communities, there was no difference in microbial composition between CON and P-EOSIS regardless of surgical intervention or the use of OCPs (Fig 2E–2H). However, surgical intervention further separated the clusters of P-EOSIS from those of CON subjects in the vaginal canal (Fig 2H, unweighted p = 0.94, weighted p = 0.11). For both GI and UG microbiome compositions, we found a significant interaction between disease state and the use of OCPs. Specifically, we found that P-EOSIS receiving OCPs had significantly different bacterial communities than P-EOSIS not receiving OCPs (Fig 3A, GI tract: unweighted R^2^ = 0.02, p = 0.002, weighted R^2^ = 0.02, p = 0.13; Fig 3B, UG tract: unweighted R^2^ = 0.01, p = 0.06, weighted R^2^ = 0.02, p = 0.01). Distance metrics, indicated by colored circles in Fig 3A and 3B, for both GI and UG tracts were larger in P-EOSIS, and OCPs enhanced this effect.

Lastly, to study if an alteration in GI/UG microbiome composition changes the concentration of urinary estrogens and its metabolites, we performed constrained analysis of principal coordinates (CAP) for bacteria in GI/UG samples with urinary estrogen metabolites included as predictor variables. Only GI tract bacterial communities were influenced by levels of estrogen and its metabolites (Table 2, CAP model: estradiol p = 0.02, 16-keto-17β-estradiol p = 0.024, estriol p = 0.035, 2-hydroxyestradiol p = 0.04, 2-hydroxylestrone p = 0.01). These results have shown that the bacterial diversity in both sites (gut/vagina) was different in P-EOSIS compared to that of CON subjects. Moreover, OCP utilization had an effect on the GI composition of both study groups.

**Table 2:** Constrained analysis of principal coordinates of GI/UG bacterial communities and the levels of estrogen and its metabolites.

	Gut Microbial					Vagina Microbial			
Source of variation	DF	Sum of Sqs	Residual	F	P value	Sum of Sqs	Residual	F	P value
Estradiol	4	1.91	11.88	1.12	***0*.*021***	1.79	12.52	0.97	0.83
16keto-17β-estradiol	4	1.88	17.06	1.13	***0*.*024***	1.78	18.79	0.97	0.74
Estriol	4	1.87	17.03	1.1	***0*.*035***	1.72	18.43	0.93	0.97
2-hydroxyestradiol	4	1.89	19.14	1.1	***0*.*036***	1.79	20.97	0.98	0.71
2-hydroxyestrone	4	1.95	19.66	1.14	***0*.*01***	1.74	21.20	0.95	0.92

Statistically significant explanatory variables are in bold and italic.

### GI/UG bacterial diversity between patients with endometriosis and controls

To assess factors (disease endometriosis, surgical intervention and hormonal therapy) driving the differences in GI/UG bacterial community compositions (species richness and uniformity), we performed alpha-diversity analysis with PERMANOVA for Simpson’s evenness, Simpson’s diversity and Faith’s phylogenetic diversity. Overall, there was an interactive impact of surgical intervention (DOS vs. PSI) and OCP utilization on bacterial alpha diversity in the GI tract (Simpson’s diversity [species richness], p = 0.034). Urogenital bacterial alpha diversity was reduced in P-EOSIS without OCPs, and an increase in alpha diversity for P-EOSIS with OCP utilization (Simpson’s diversity, p = 0.01). Bacterial diversity was lower in P-EOSIS without OCPs, whereas diversity was greater in P-EOSIS receiving OCPs (Simpson’s diversity, p = 0.042). Microbial alpha diversity in GI/UG did not correlate with specific estrogen and estrogen metabolites in the urine of our subjects (S1A & S1B Fig). These results indicate that surgical intervention and hormonal therapy drives the differences in species richness of GI/UG bacterial community compositions.

### Taxonomic variation in gut and vaginal microbiota in subjects with endometriosis

**Fig 4:**
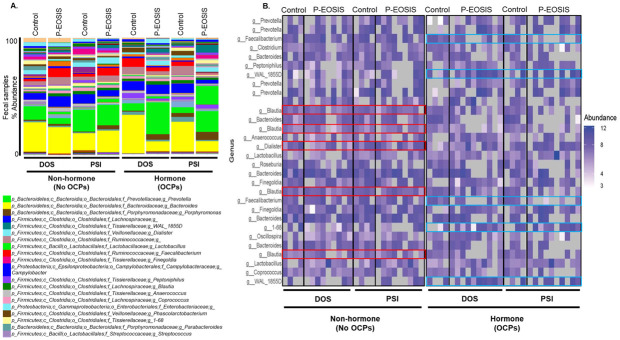
Relative abundance of GI microbial dynamics in control subjects and patients with endometriosis (P-EOSIS). Subjects were either not receiving hormonal therapy (OCPs) (control = 4; P-EOSIS = 9) or receiving OCPs (control = 4; P-EOSIS = 10). **A**. Level 6 (genera) taxonomical summary plots for fecal samples. Samples were collected from subjects at the time of surgery (DOS) (left panel) and following surgical intervention (PSI) (right panel). **B**. The top 30 abundant bacterial genera in the GI tract of control and P-EOSIS.

Mucosal microbiota composition was altered in P-EOSIS at DOS and PSI in both GI/UG. Regardless of hormonal intervention, *Firmicute* species were more abundant in P-EOSIS (compared to CON) at both study time points (Fig 4). The most abundant phylum identified from fecal samples was *Firmicutes* (without OCPs: DOS: 44.7% control, 55.7% endometriosis; PSI: 52.8% control, 50.6% endometriosis; with OCPs: DOS: 41.8% control, 43.8% endometriosis; PSI: 45.3% control, 52.7% endometriosis), followed by *Bacteroidetes* (without OCPs: DOS: 40.1% control, 33.7% endometriosis; PSI: 35.9% control, 42.4% endometriosis; with OCPs: DOS: 51.7% control, 45.2% endometriosis; PSI: 47.2% control, 40.2% endometriosis). Interestingly, we also found that the GI microbial site had both shared and unique species depending on whether or not endometriosis was present on both the DOS and PSI (Table 3). Mostly, the number of unique species was higher for CON than P-EOSIS, with the exception of subjects not receiving OCPs, P-EOSIS had a higher number of unique species compared to CON at DOS (Table 3, CON = 13,883, P-EOSIS = 16,013).

**Table 3:** Comparison of fecal and vaginal phylotypes samples between controls and patients with endometriosis.

Hormone treatment	Fecal				Vaginal		
	Sample	Unique-Control	Shared	Unique- Disease	Unique-Control	Shared	Unique- Disease
No	DOS	13,883	14,697	16,013	4,631	2,749	4,903
	PSI	18,813	13,707	12,048	4,685	2,615	4,599
Yes	DOS	18,777	13,603	10,965	4,477	2,963	4,689
	PSI	16,785	10,695	9,909	4,737	2,663	4,677

Summary of phylotypes identified within each sample type characterized by if they are **unique** or **shared** between controls or patients with endometriosis.

At the genus level, the GI bacterial communities of subjects receiving OCPs were dominated by *Bacteroides*; this was followed by *Prevotella*, *Blautia*, *Faecalibacterium*, *Dialister*, *Coprococcus*, *Faecaliobacterium*, and *Sutterella* (Fig 4B). Surgical intervention alone (no OCPs) increased the levels of *Blautia* and *Dialister* in P-EOSIS, but decreased the overall abundance of other genera in the GI of both study groups (Fig 4B, red box). For subjects who utilized OCPs, we observed an increase in the *1–68* and *WAL_1855D* genera, but a decrease in *Faecalibacterium* in their GI communities (Fig 4B right panel, blue box). *Bacteroides* were still dominant in CON subjects receiving OCPs but lower in P-EOSIS receiving OCPs at DOS (Fig 4B right panel). Surgical intervention in conjunction with OCP use once again altered the abundance of GI communities in P-EOSIS and CON subjects (Fig 4B).

**Fig 5:**
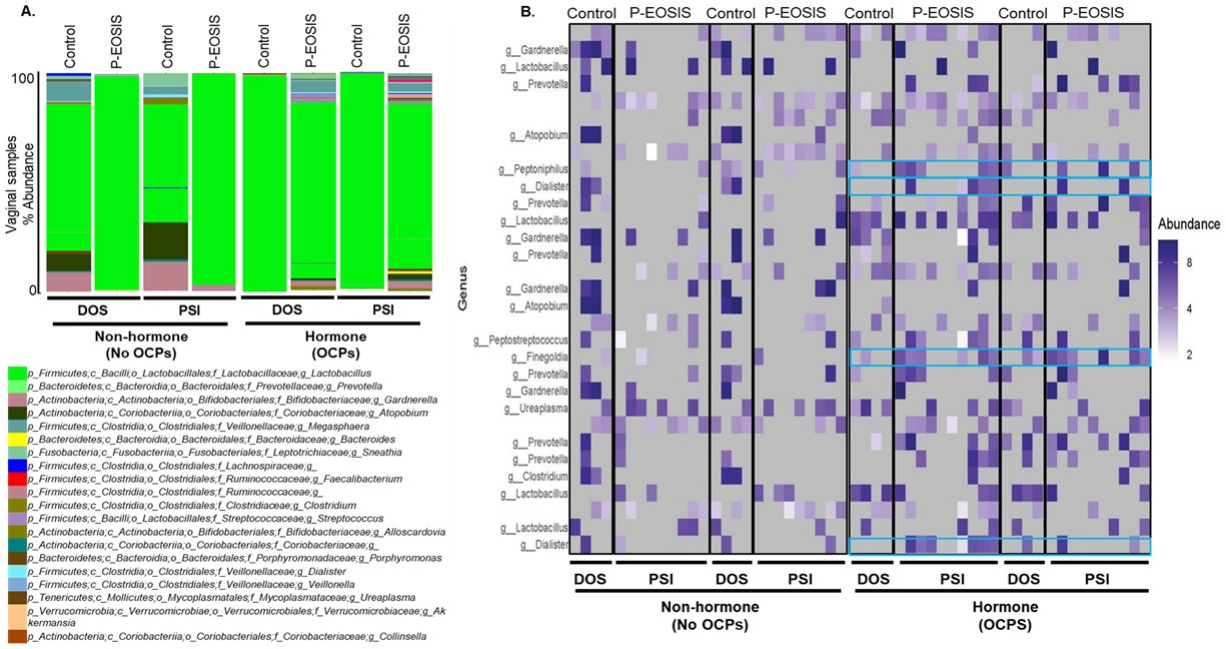
Relative abundance of vaginal microbial dynamics for control patients and patients with endometriosis (P-EOSIS). Subjects were either without hormonal therapy (control = 4; P-EOSIS = 9), or on hormonal therapy (control = 4; P-EOSIS = 10). **A**. Level 6 (genera) taxonomical summary plots for vaginal samples. Samples were collected from subjects on the day of surgery (DOS) (left panel) and following surgical intervention (PSI) (right panel). **B**. The top 30 abundant bacterial genera in the UG tract of control and P-EOSIS.

Similarly, for the vaginal tract P-EOSIS exhibited microbial shifts between the vaginal samples taken on the DOS and those collected at PSI regardless of hormonal suppression (Fig 5A). *Firmicutes* was the predominant phylum (without OCPs: DOS: 69.8% control, 99% endometriosis; PSI: 47.2% control, 97% endometriosis; with OCPs: DOS: 99% control, 84.9% endometriosis; PSI: 97.7% control, 74.7% endometriosis), followed by *Actinobacteria* (without OCPs: DOS: 17.2% control, 0.2% endometriosis; PSI: 31.4% control, 2.6% endometriosis; with OCPs: DOS: 0.2% control, 5.2% endometriosis; PSI: 0% control, 7.9% endometriosis). Regardless of whether endometriosis was present, both shared and unique species were found in the vaginal tract at the DOS and PSI (Table 3, right panel). Mostly, the number of unique species was higher for P-EOSIS than CON, and surgical intervention reduced number of unique species in P-EOSIS regardless of the use of hormonal therapy (Table 3, without OCPs: DOS: 4,903; PSI: 4,599; with OCPs: DOS: 4,689; PSI: 4,677).

At the genus level, the UG bacterial communities of subjects not receiving OCPs were dominated by *Lactobacillus*, and followed by *Gardnerella*, *Prevotella*, *Atopobium*, *Clostridium* and *Dialister* (Fig 5A and 5B). In the absence of OCPs, P-EOSIS had higher levels of *Lactobacillus* but lower levels of other genera in comparison to CON. Surgery only (without OCPs) altered the abundance of all genera in the vaginal tract of both study groups (Fig 5B left panel). For P-EOSIS who received OCPs, we observed an increase in the following genera in their UG communities: *Peptoniphilus*, *Dialister*, *Finegoldia* and *Ureaplasma* (Fig 5B right panel, blue box). *Lactobacillus* was decreased in P-EOSIS who received OCPs, but increased in CON who received OCPs at DOS and PSI (Fig 5B right panel). Once again surgical intervention and hormonal therapy altered the abundance of vaginal bacterial communities in both study groups (Fig 5B).

Next, P-EOSIS had a higher ratio of *Firmicute/Bacteroidetes* compared to CON (in GI tract: 1 in CON, 1.2 in P-EOSIS; in vaginal tract: 10.6 in CON, 14.2 in P-EOSIS) regardless of surgical intervention or hormonal therapy. These results indicate a dysbiosis within the gut and in the vaginal tract of P-EOSIS, and the presence of endometriotic lesions altered not just the quantity of each microbial species that was present, but also the composition of microbial species that were present.

### Association of GI/UG microbiome and the levels of urinary estrogens in patients with endometriosis and controls

**Fig 6:**
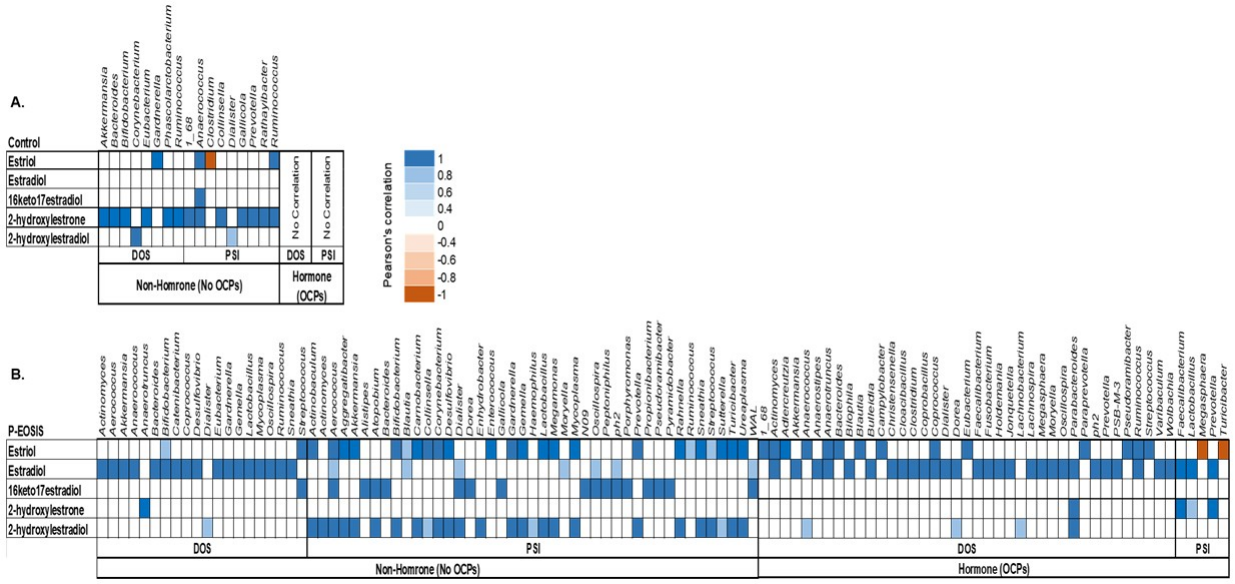
Pearson’s correlation coefficient between GI bacterial communities and the levels of urinary estrogens and their metabolites. All subjects were either receiving or not receiving hormonal treatment (OCPs). **A**. Control patients; **B**. Patients with endometriosis (P-EOSIS).

**Fig 7:**
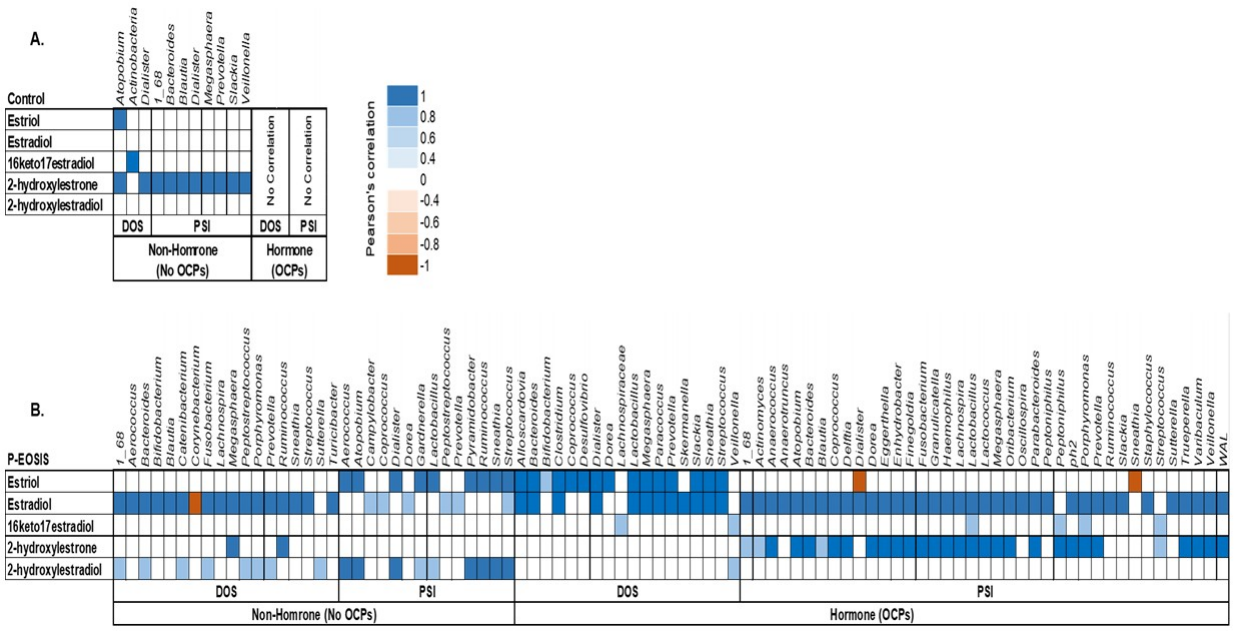
Pearson’s correlation coefficient between UG bacterial communities and level of urinary estrogens and its metabolites. All subjects were either receiving or not receiving hormonal treatment (OCPs). **A**. Control patients; **B**. Patients with endometriosis (P-EOSIS).

To investigate the relationship between the GI/UG bacteria species and the concentrations of urinary estrogen and its metabolites, we performed Pearson’s correlation coefficient for each bacterial site (GI, UG) with the concentration of 15 urinary estrogens. Bacterial phyla (*Actinobacteria*, *Bacteroidetes*, *Firmicutes*, *Fusobacteria*, *Proteobacteria*, *Synergistetes*, *and Verrucomicrobia*) in GI and UG were correlated with concentrations of 5 metabolites: 17β-Estradiol (E2), estriol (E3), 2-hydroxyestradiol, 2-hydroxyestrone, and 16-keto-17β-estradiol (Figs 6 & 7). Mostly, these phyla increased in abundance with increased concentration of estrogen and its metabolite for both study groups (CON vs. P-EOSIS).

In the GI tract, the correlation between level of urinary estrogens/metabolites and the abundance of gut bacteria were affected by the presence of endometriosis (Fig 6). P-EOSIS had a strong association on gut bacteria with levels of urinary E2, E3, 2-hydroxyestradiol, and 16-keto-17β-estradiol (Fig 6B); while CON subjects had positive correlation between gut bacteria and the concentration of urinary 2-hydroxyestrone (Fig 6A). Surgical intervention also impacted the relationship between GI bacterial species and the concentrations of urinary estrogens/metabolites in P-EOSIS. In P-EOSIS, the gut bacteria was positively correlated with the concentration of E2 at DOS, and a positive correlation with the concentrations of urinary E3, 16-keto-17β-estradiol and 2-hydroxyestradiol after surgical intervention (no OCPs) (Fig 6B, right panel). We detected a higher number of GI bacteria that correlated with urinary estrogens/metabolites at PSI compared to DOS for P-EOSIS not utilizing OCPs (Fig 6B, right panel). Likewise, hormonal therapy affected the relationship between the GI bacteria species and the concentrations of urinary estrogens/metabolites in both study groups (Fig 6). In P-EOSIS, OCP use resulted a higher amount of gut bacteria that correlated with estrogen/metabolites at DOS, while hormonal therapy and surgical intervention decreased number of bacteria that correlated with urinary estrogens/metabolites (Fig 6B). No correlation was noted between GI species and the urinary estrogens/metabolites for CON (Fig 6A). Together, these data indicate abnormal levels of urinary estrogens and its metabolites were highly correlated with altered GI bacterial composition in P-EOSIS. The biliary excretion of estrogens and the enterohepatic circulation of estrogen metabolites appears to be crucial in the alteration of GI microbial communities that are distinct from those of non-diseased patients: the concentrations of excreted E2, E3, 16-keto-17β-estradiol and 2-hydroxyestradiol (conjugated estrogens) mostly were lower in P-EOSIS, which indicates a higher level of bioactive estrogens/metabolites in the circulation of P-EOSIS. This could be the source that is driving changes in GI tract of these subjects.

Similar to results of the GI tract, in the UG tract, the correlation between levels of urinary estrogens/metabolites and the abundance of vaginal bacteria were affected by the presence of endometriosis (Fig 7). In CON subjects, we observed an association between UG bacteria and the concentration of urinary 2-hydroxyestrone (Fig 7A); in PEOSIS, a strong correlation was found between UG bacteria and levels of urinary E2, E3, 2-hydroxyestradiol, and 2-hydroxyestrone (Fig 7B). Surgical intervention had no effect on the CON group (Fig 7A). However in P-EOSIS, a positive correlation was detected between bacteria in the vaginal canal and the concentration of E2 and 2-hydroxyestradiol at DOS. The correlation of UG microbiota and estrogen/metabolites was enhanced by additional correlation of urinary E3 with UG bacterial species after surgical intervention (no OCPs) (Fig 7B, right panel). Finally, OCP use also affected the relationship between the UG bacterial species and the concentrations of urinary estrogens/metabolites in P-EOSIS (Fig 7). We observed a higher number of UG bacteria that associated with urinary estrogens/metabolites at PSI compared to DOS for P-EOSIS with OCPs (Fig 7B, right panel). These results showed that aberrant levels of urinary estrogens and its metabolites were highly correlated with an altered UG bacterial composition in P-EOSIS. Particularly, the altering in concentrations of E2 and E3 could influence the alteration of the UG microbiome community in these patients.

In summary, both surgical intervention and hormonal therapy altered bacterial communities within GI and UG tracts of P-EOSIS. Specifically, the dysbiosis of GI/UG microbial communities associated with aberrant levels of estrogen and its metabolites.

## Discussion

Commensal bacterial species in the gut and the female reproductive tract play a major role in the maintenance of the endocrine system and are concomitantly influenced by various host factors such as immunological response, metabolic changes and the environment [36]. Thus, a shift in the commensal microbial community in the gut and the reproductive tracts are indicative of a potential shift in hormone synthesis and signaling pathways. In this study, we found that microbial dynamics of P-EOSIS were altered compared to those of non-diseased subjects. Still, restoration of healthy microbial communities in P-EOSIS was not achieved following surgical intervention. Additionally, estrogen metabolite levels in P-EOSIS were also abnormal. However, due to the reduced number of subjects, a conclusion regarding the relationship between the microbiome and estrogen metabolism in P-EOSIS may be limited in this particular study and warrants further validation.

Our first goal was to determine the levels of endogenous estrogen and estrogen metabolites in P-EOSIS and CON, and investigate potential effects of OCPs on these levels. Endometriosis is an estrogen-dependent disease [1]. As estrogen is a mitogen that increases mammary epithelial and stromal cell growth [37], higher systemic levels may mediate the process of attachment and the survival of endometrial fragments within the peritoneal cavity [38]. Very little is known regarding the type and quantity of endogenous estrogens and their metabolites in urine samples from P-EOSIS. In this study, we detected higher levels of 17β-estradiol and 16-keto-17β-estradiol in P-EOSIS regardless of the use of OCPs. Lower levels of estriol, 2-hydroxyestrone and 2-hydroxyestradiol were observed in P-EOSIS, predominantly in those who were utilizing OCPs. Thus, 16-keto-17β-estradiol and 2-hydroxyestradiol of the 2-OH pathway were the most affected by the presence of endometriosis depending on the usage of hormonal suppression therapy and surgical interventions. To date, the only two published studies investigating the role of estrogen metabolites in endometriosis concluded that 2-hydroxyestradiol and 2-methoxyestradiol reduced endometriotic cell growth via estrogen receptor independent mechanisms [39]; and endometriosis metabolizes estrogen preferentially to the biologically active of 2-hydroxyestradiol, and genotoxic 4-hydroxyestradiol and 4-hydroxyestrone metabolites [40]. Various reports have also indicated that prolonged exposure of target tissues or cells to excessive estrogens is an important etiological factor for the induction of estrogen-associated cancers in experimental animals [41], and in humans [42–44]. However, the role of these endogenous estrogens and estrogen metabolites in endometriosis is still unclear and further research is needed.

Our second goal was to define the composition of the GI and UG microbiomes in P-EOSIS and identify their potential variations associated with this disease. The GI microbiome diversity is crucial in health maintenance as microbiota and their metabolites have been proven to play a fundamental role in estrogen production and signaling [45, 46]. In this regard, the gut microbial communities secrete β-glucuronidase, an enzyme that deconjugates estrogen, allowing it to bind to estrogen receptors, leading to its subsequent physiological downstream effects. A reduction in commensal microbial diversity due to dysbiosis and inflammation reduces β-glucuronidase activity which might result in altered estrogen signaling. We observed that the GI bacterial communities had higher diversity and richness than the UG bacterial communities in both study cohorts regardless of surgical intervention. Consistent with previous studies, more than 80% of all study subjects’ microbiota were composed of *Actinobacteria*, *Bacteroidetes and Firmicutes* [20, 47, 48]. Unlike the GI microbiome of control subjects, where the predominant species (such as *Lactobacillus* and *Bifidobacterium infantis*) promote immune-tolerance, bacterial communities present in the gut of P-EOSIS promote inflammation. An elevated ratio of Firmicutes/Bacteroidetes has been associated with obesity [49, 50], colorectal cancer [51], rheumatoid arthritis [52] and IBD disorders [53]. A recent review from Magne F. et al. suggested that using Firmicutes/Bacteroidetes ratio for determination of health status would be a challenge due to multiple discrepancies such as lifestyle associated factors and sampling process. In this study, we observed the ratio of Firmicute/Bacteroidetes in P-EOSIS to be higher than that in CON subjects. The reduction in the levels of *Bacteroides* and *Prevotella* spp. (phylum of *Bacteroidetes*) and high levels of Clostridium spp. (Firmicutes phylum) observed in P-EOSIS may play a role in endometriosis associated inflammation by induction of colonic Foxp3^+^ regulatory T cells and activation of T cell dependent immunoglobulin A production [54–56]. Our results indicate a dysbiosis in the GI and UG microbiomes of P-EOSIS concomitant with alterations in the production of estrogen and its metabolites. Our findings are supported by the recently published study by Shan et al., where they elucidated the associations between gut microbial species and serum hormones and inflammatory cytokines [57]. However, the mechanism(s) of action between specific GI/UG species and estrogen metabolism in endometriosis still need further investigation.

Finally, we investigated if surgical intervention could restore microbial homeostasis as well as estrogen metabolism in P-EOSIS. Surgery is currently the most successful treatment option for women with endometriosis who are hoping to achieve a spontaneous pregnancy [7, 58]. LC-MS/MS analysis showed an impact of surgery on P-EOSIS estrogen metabolism (17β-estradiol and 2-hydroxyestradiol). We also observed that microbial community profiles were altered after surgical intervention for all subjects analyzed. In our assessment of the intra-subject effects of surgical intervention over time, the level 6 taxon (genus) summary revealed a change in microbial communities, indicating that surgery influences the microbial community dynamics of P-EOSIS. Women with endometriosis have an increased likelihood of pregnancy by 73% within 36 weeks of surgical intervention [59, 60]. However, surgery is still an invasive procedure, and is not effective permanently since up to 36% of patients will require further surgery after 2 years [59, 60]. Together, this indicates that surgical intervention, as a sole treatment strategy, may not be effective for all endometriosis patients; therefore, further investigation into alternative treatments for these patients is necessary.

In summary, our findings provide evidence that there may be a unique microbiome “signature” in the GI and UG tracts, as well as a distinct estrogen metabolite profile in P-EOSIS. The major findings of this study are: 1) the mucosal microbiomes (GI and UG) exhibited a unique profile in P-EOSIS vs. CON; 2) levels of endogenous estrogen and estrogen metabolites were altered in urine samples of P-EOSIS; 3) OCP treatment altered bacteria populations in the gut and vaginal canal of P-EOSIS; 4) surgical intervention resulted in microbial shifts between the DOS and PSI time points; and 5) increased post-surgical variability in microbial community dynamics was noted for P-EOSIS. Our results suggest that a unique profile associated with endometriosis may be utilized as a clinical tool for diagnosis of the disease, potentially eliminating the need for invasive laparoscopic surgery for diagnostic purposes; thus, allowing women to be diagnosed sooner and begin treatment earlier in the disease process. Further investigations into microbial shifts associated with endometriosis staging is warranted.

### Strengths and limitations

Our findings support additional investigation to further elucidate the specificity of the microbial dysbiosis observed in P-EOSIS and the long-term effect of endometriosis pathogenesis on these physiological systems using experimental animal models. Moreover, the analytical approaches utilized in this work open the field for future investigations related to microbial function in patients with endometriosis through metabolomics and multi-omics analyses that will ultimately provide relevant mechanistic information on the pathogenesis and progression of the disease, endometriosis. The main limitation of this study was that analyses for subjects with different stages of endometriosis was not performed due to the small sample size.

## Supporting information

S1 Fig Alpha-diversity rarefaction curves of bacterial operational taxonomic units of control subjects and patients with endometriosis (P-EOSIS). Phylogenetic diversity rarefaction curves of bacterial OTUs from fecal specimens and vaginal samples of all subjects were analyzed by Qiime2. This is based on Faith’s phylogenetic diversity and the curves represent the mean diversity indices for each sample. **A**. Fecal samples. **B**. Vaginal samples.
(TIF)

S1 File (ZIP)
