# Supplementary figures and images for: Association of microbial dynamics with urinary estrogens and estrogen metabolites in patients with endometriosis
Source: PLoS One. 2021 Dec 16;16(12):e0261362. doi: 10.1371/journal.pone.0261362 (PMC8675749; doi:10.1371/journal.pone.0261362)

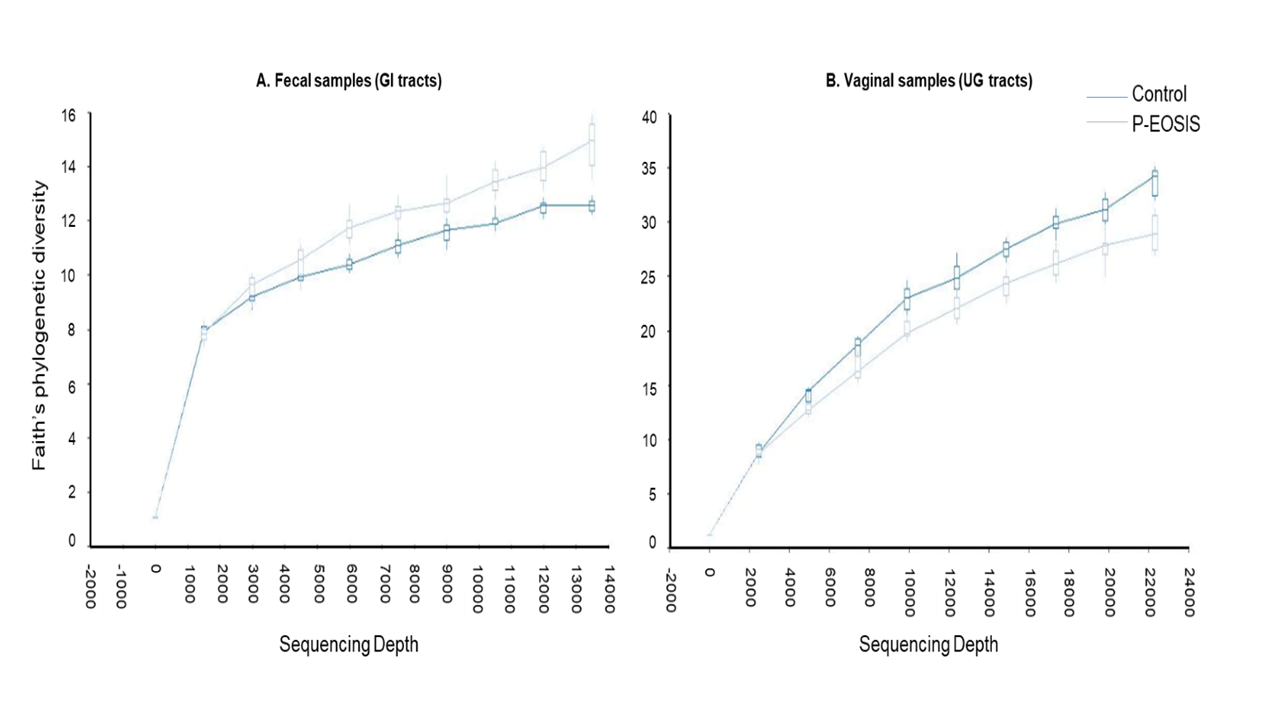

Supplement: S1 Fig — Phylogenetic diversity rarefaction curves of bacterial OTUs from fecal specimens and vaginal samples of all subjects were analyzed by Qiime2. This is based on Faith’s phylogenetic diversity and the curves represent the mean diversity indices for each sample. A. Fecal samples. B. Vaginal samples. (TIF) [file pone.0261362.s001.tif]
